# The Limits of Humans in Data Gathering: Documentation Error Rates in the Electronic Health Record in the Operating Room

**DOI:** 10.1007/s10916-026-02346-9

**Published:** 2026-02-09

**Authors:** Andrew R. Bradley, Abner Barbosa, Logan Younk, Naila Rocha, Peter F. Nichol

**Affiliations:** 1 Department of Surgery, University of Wisconsin School of MedicineUniversity of Wisconsin School of Medicine, and Public Health, H4/746 CSC 600 Highland Ave, Madison, WI 53792 USA; 2 Department of Animal and Dairy Sciences, University of Wisconsin- Madison, Madison, WI USA

## Abstract

We studied the accuracy of nursing electronic health record (EHR) documentation during surgical procedures focusing on time-stamping of events. In high-end manufacturing these tasks would be considered simple routine with error rates of 0.1% to 0.5%. Documentation accuracy of six key events over 10 consecutive weeks was measured. Error rates, delays in documentation, and environmental variables including operating room (OR) size, number of nurses per OR, and service line were recorded. Statistical methods included regression models, correlation analysis and analysis of variance (ANOVA). From 202 cases, 98.10% of 1240 events were captured, revealing an overall error rate of 17.01%. Delays in documentation were observed in 51.40% of events. Larger ORs, multiple nurses documenting, and delays over 20 min were linked to higher error rates. Error rates increased from 1.30% without delays to 38.43% with delays exceeding 20 min. Documentation error rates for time-stamping tasks were at a minimum 2.6 times higher than predicted and increased significantly with delays in documentation indicating that environmental complexity and stress diminish human tasks performance as well as recall of events. Our data indicate that error rates in the healthcare space are higher than in the manufacturing space for tasks of identical or similar complexity. Given the certainty and immutability of human error in task performance, our findings highlight the need for technologies to improve accuracy of EHR documentation in this critical and high complexity space.

**Supplementary Information:**

The online version contains supplementary material available at 10.1007/s10916-026-02346-9.

## Objective

### Background

Surgical Operating Room (OR) informatics are generated from data entered into the Electronic Health Record (EHR), and these data serve as the foundation for clinical decision-making, quality improvement, medico-legal documentation, billing, and research. Most of these data originate from human entry, which is vulnerable to error [1]. One of the most comprehensive texts on this topic is Smith’s *Reliability*,* Maintainability and Risk* [2] which provides detailed analyses of error rates associated with specific human tasks. According to Smith, two key variables affect error rates: task complexity and environmental stress. Smith categorizes tasks into four groups of escalating complexity: simplest possible, routine simple, routine with care, and complicated non-routine. Error rates increase logarithmically with task complexity, ranging from 0.01% for the simplest tasks to anywhere from 10% to 90% for complicated, non-routine tasks.

Awareness of errors in healthcare has grown since publication of the National Academy of Medicine’s treatise *To Err is Human* [3] which estimated that 98,000 people die from medical errors in hospitals annually. This prompted a focus on improving safety in healthcare. One of the most well-known outcomes of this focus was introduction of the pre-incision time-out as part of the Universal Protocol by the Joint Commission in 2003 [4] in an effort to prevent wrong site, wrong procedure, wrong person surgeries. More recent studies indicate that the OR continues to be a high-risk environment for errors which impairs both accuracy of communication and patient safety [5, 6].

Although little research has examined documentation error rates within surgical nursing workflows, the anesthesiology literature provides important parallels. Avidan et al. (2014) demonstrated substantial inaccuracies in manually entered drug-administration times, highlighting that anesthesiology documentation, like OR nursing documentation, is highly susceptible to time-stamp errors when clinicians are engaged in simultaneous clinical tasks [7].

Further, Grundgeiger et al. (2024) showed that documentation behavior among anesthesiologists is strongly driven by medico-legal motives and the need to preserve a coherent narrative, rather than the need for real-time precision [8]. As a result, delays and retrospective entries are common and often intentional. These findings underscore that documentation accuracy cannot be understood solely as a technical recording task; it is shaped by user motivation, workflow, perceived liability, and cognitive demands.

Similar concerns have emerged in surgical workflows. Nichol et al. (2023) showed that staff-reported errors with surgical instruments occurred at only 2.6% of the actual rate measured by dedicated observers. Moreover, staff-reported data showed significant shortcomings, with 79.6% of reports left incomplete and 15.4% delayed by more than a day [9]. Yet these staff-reported data were being used to assess OR safety and efficiency and to identify breakdowns in the system for reprocessing surgical instruments [10].

Building on these insights, recent work has aimed to design improved documentation tools. For example, Huber et al. (2025) evaluated an AI-based documentation assistant that supported anesthesiology teams and demonstrated reductions in cognitive burden and improved temporal coherence of documentation [11].

Despite clear parallels between surgical nursing and anesthesiology workflows, little is known about how environmental complexity, workflow delays, and multitasking influence OR time-stamping accuracy. Our study addresses this gap by empirically evaluating documentation error rates across six key intraoperative events.

#### Significance

We set out to measure the accuracy of EHR nurse documentation during surgery and determine the frequency and variables that contribute to documentation errors. For the purposes of this study, we focused on the simplest charting task: time-stamping. We studied 6 events. The first two (patient in the room and pre-procedure verification) happen prior to the start of the procedure when the patient is awake, and the maximum number of staff are in the room who are frequent multi-tasking. The next two (final timeout and incision) occur at the beginning of the procedure when the patient is asleep and fewer staff are present. Next, final wound closure/completion of procedure occurs typically when staff are completing soft and hard counts of instruments and devices. Finally, there is patient out of the room in which the procedure and all tasks are complete, the patient is on a bed and stable and the only thing to do is leave the OR. We then set out to determine the following: (1) Whether time-stamping a seemingly “simple routine” task based on comparisons to similar tasks in Smith’s *Reliability*,* Maintainability and Risk* (7th Edition) has the predicted error rate of a simple routine task (0.1% to 0.5%) [2]? (2) Whether time-stamping events occurring during periods of increased environmental complexity [12] have higher error rates in documentation (service lines with more equipment, more circulating nurses in the room, more people in the room, multiple service line cases etc.)? And finally, (3) Whether delays in documentation are associated with higher rates of error? We based this last query on data that indicate cognitive loading in complex environments diminishes memory recall [1].

## Materials and Methods

Human ethics approval and consent to participate were not applicable. This study received an exemption from the University of Wisconsin Institutional Review Board as it was a quality improvement project. Observations were conducted over 10 weeks during the summer of 2023 at our system’s Children’s Hospital, a 111-bed facility connected to a larger medical center. The operating room suite has eight operating rooms, with elective hours from 7:30 a.m. to 5:00 p.m., Monday through Friday, except Wednesdays, when the start time is 8:30 a.m. Each OR features a nurses’ station with an ergonomic desk, chair, and large-screen desktop computer, positioned to the left of the OR doors with a clear view of the OR table. The healthcare system uses the Epic Systems EHR (Verona, WI).

## Observation Process

Two observers conducted 19 beta observations to familiarize themselves with nursing workflows, EHR charting, and optimal positioning within the OR. These beta observations refined methods for data collecting on six time-stamping events and recording when nurses documented these events in the EHR. The time-stamping events were: patient in the room (In OR), pre-procedure verification (Pre-Proc Ver), final timeout (Time out), incision (Incision), final wound closure/completion of procedure (Closure), and patient out of the room (Out of OR). Observers recorded whether charting of all 6 tasks were completed before the patient left the OR. Formal data collection commenced after the beta observations.

For each designated event, observers recorded the time of occurrence and compared it with the time documented in the EHR. Data were stored in Google spreadsheets. Initially, both observers worked together to verify consistency across their observations for 60 cases. Once inter-rater reliability was confirmed (concordance > 99%), remaining observations were conducted by a single observer per room.

Nursing staff were informed of the project one month prior to its initiation and were aware of observers’ presence during the study period. However, nurses were instructed not to consult observers regarding event timings and remained blinded to the study’s findings throughout. Additionally, the number of nurses per room responsible for time-stamping was recorded, along with the frequency of instances where nurses had to leave the computer for other activities during the surgery.

Nine surgical service lines were assessed: Dental (DEN), Otolaryngology (OTO), General Surgery (GEN), Gastroenterology (GI), Neurosurgery (NEURO), Ophthalmology (OPH), Orthopedics (ORTH), Plastic Surgery (PLA) and Urology (URO), as well as cases involving multiple service lines termed Multi-Panel (M.P.) procedures. For single-panel (single service line) cases, the total number of clickable events was calculated by counting the total clicks required from EHR in drop down menus during five representative surgeries. The hospital’s total annual case volume was also determined by analyzing the number of cases completed during the first 11 months of 2024.

### Data Analysis

Statistical analyses were performed to evaluate delay and error rates. Observed errors were defined as any event that was documented with more than a 60-second difference from when it occurred, and were summarized as percentages of total events. Error distributions were assessed using the Shapiro-Wilk test to guide the selection of parametric or non-parametric statistical methods. Chi-square tests examined associations between categorical variables, such as nurses/OR and error for event type, and identified significant differences in group error rates. Pearson’s correlation coefficient measured associations between delay rates and error rates, and a simple linear regression model quantified the relationship’s strength and direction. Analysis of variance (ANOVA) was conducted to compare mean error rates across groups stratified by procedure length and nurse interruptions. Logistic regression models were constructed to predict the likelihood of errors at various surgical stages. Predictor variables included OR size, nurses/OR, procedure length, delays, interruptions, and service line. Logistic regression outputs included coefficient estimates, odds ratios, and 95% confidence intervals to assess predictors’ influence on error occurrence.

This methodology allowed for a comprehensive evaluation of error rates across surgical stages and the identification of contributing factors. Key surgical events were analyzed to compute error rates, facilitating group-wise comparisons and the exploration of predictors through advanced statistical models.

## Results

Data on 1,217 out of a possible total of 1,240 events were captured yielding a data capture rate of 98.10%. Event capture rates were 99.50% for ‘In OR’, 100% for ‘Pre-Procedure Verification’, 97.69% for ‘Timeout’, 99.50% for ‘Incision’, 92.08% for ‘Closure’, and 100% for ‘Out of OR’ timestamps (Table 1). On average, each single-panel case in the EHR involved 253 clickable, documentable events.

The minimum overall error rate for any of the six events studied was 9.95%. This exceeded the expected error rate of 0.1% to 0.5% for a simple routine task. This can be interpreted in one of several ways. Either the task of time-stamping in the EHR is far more complex that we understood it to be or the complexity and/or stress in the environment influences performance of these tasks to such a degree that it assumes the performance metrics of a complicated non-routine task with an error rate at or above 10%.

The analysis of EHR documentation by event type revealed differences in the accuracy, delay rates, and error rates across distinct procedural milestones. 1,010 events (82.99%) were correctly documented, with varying levels of errors among event types. The ‘Pre-Procedure Verification’ had the highest error rate at 27.31%, followed by ‘Out of OR’ at 20.30%. ‘Timeout’ and ‘Closure’ had moderate error rates at 14.69% and 15.05%, respectively. The lowest error rates were observed in ‘In-OR’ (13.93%) and ‘Incision’ (9.95%) (Table 1).

Documentation delays were observed in 630 events, 51.40% of the total, with significant variability across event types. The highest delay rates were found in ‘Pre-Procedure Verification’ (97.87%) and ‘Timeout’ (81.42%), both of which occur during the busiest phases of the surgery and involve multiple team members who are frequently multi-tasking. In contrast, ‘Closure’ and ‘Out of OR’ had lower delay rates at 22.09% and 20.74%, respectively. The average length of delays for procedures with recorded delays varied significantly, with the longest average delays occurring during ‘Pre-Procedure Verification’ (25.25 min) and ‘In OR’ (23.92 min). In contrast, ‘Closure’ and ‘Incision’ experienced shorter delays, averaging 5.85 min and 5.18 min, respectively. These findings underscore the relationship between the complexity and activity levels of different procedural events and the percentage and duration of delays.

**Table 1:** Results by event Type. Documentation accuracy and delays in EHR time-stamping by event type. Metrics include the total number of events, the percentage of events correctly recorded, delays (frequency and average duration in minutes), and error rates. The average delay for “Out of OR” events could not be calculated, as nurses frequently documented this event outside the OR after patient had left and the observers were unable to discern when Documentation occurred

Metric	In OR	Pre-Proc Ver	Timeout	Incision	Closure	Out of OR	Total
Events	201	216	211	201	186	202	1217
Events documented accurately in EHR	173	157	180	181	158	161	1010
Delays	80	207	173	84	45	41	630
% with delays	41.18%	97.87%	81.42%	43.32%	22.09%	20.74%	51.40%
Average Length of Delay in Minutes (Standard Deviation)	23.92 (16.07)	25.25 (16.41)	6.80 (7.07)	5.18 (5.19)	5.85 (3.97)	-	13.40 (9.74)
Error Rate	13.93%	27.31%	14.69%	9.95%	15.05%	20.30%	17.01%

The chi-square analysis of error rates by OR size revealed a statistically significant relationship to the ‘Pre-procedure Verification’ event (*p* = 0.0320), with larger ORs exhibiting higher error rates compared to smaller ones. This finding suggests that higher error rates occur in environments or periods of increased complexity. Typically, procedures performed in larger ORs involved service lines (GEN, NEURO, ORTH) that require more equipment, personnel, and intricate workflows, introducing additional complexity in these cases.

Similarly, the chi-square analysis of the number of nurses involved in charting demonstrated a statistically significant association with higher error rates during ‘Pre-procedure Verification’ event when more than one nurse was present in the room (*p* = 0.0145). In contrast, other procedural steps, including ‘In OR,’ ‘Timeout,’ ‘Incision,’ ‘Closure,’ and ‘Out of OR’ did not exhibit significant differences (*p* > 0.05). Consistent with previous studies [13], an increased number of individuals potentially sharing charting responsibilities may increase environmental complexity during the early stages of the procedure, where clear communication and task delineation are critical.

Interestingly, linear regression did not find a significant effect of surgery length on overall error rates (β = 0.00034, 95% CI [–0.00102, 0.00171], *p* = 0.387; R² = 0.375). Likewise, interruptions in nurses’ charting were also not significantly related to overall error rates (β = 0.00333, 95% CI [–0.00060, 0.00726], *p* = 0.068; R² = 0.869). However, logistic regression analyses indicated that predictors of increased error rates for the ‘Pre-procedure Verification,’ included having more than one nurse charting (OR = 3.02, 95% CI [1.14–8.01], *p* = 0.026) and procedure lengths between 11 and 20 min (OR = 0.20, 95% CI [0.044–0.946], *p* = 0.043). The former of these results suggest that additional people in the room and the latter, compression of case length when charting 253 tasks/case in EHR may also contribute to environmental complexity that increases error rates.

**Fig. 1:**
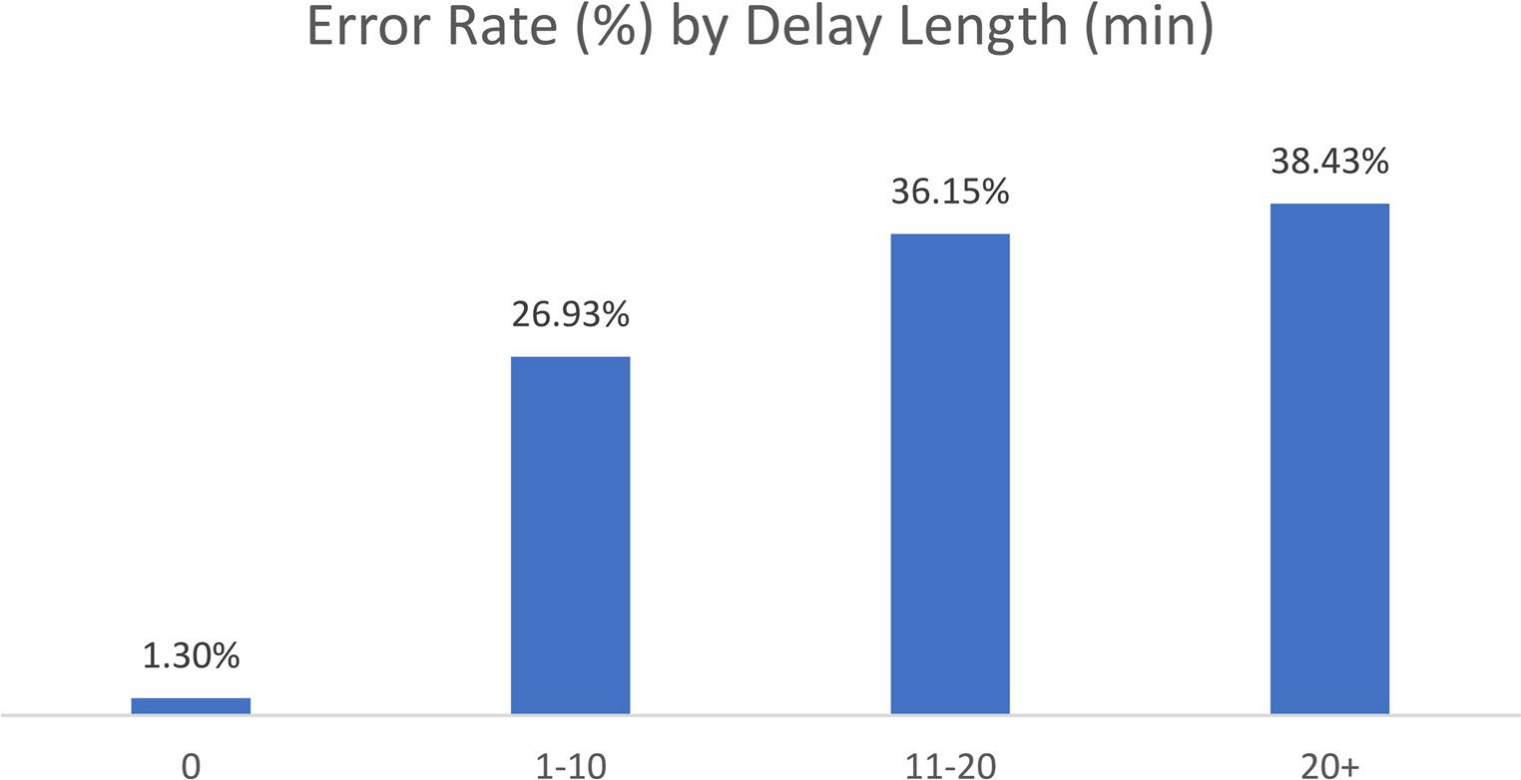
Error rate by delay length in minutes

Correlation analysis demonstrated a strong positive link between overall delay rates and overall error rates (r = 0.71). Notably, when no delay occurred between the event and its documentation, the error rate was as low as 1.30% (Fig. 1.). However, error rates escalated sharply with increasing length of delays: 26.93% for delays of 1–10 minutes, 36.15% for 11–20 minutes, and peaking at 38.43% for delays exceeding 20 minutes. Logistic regression analysis indicated that delays exceeding 20 minutes were significantly associated with increased error rates during the ‘Timeout’ event (OR = 348, 95% CI [8.6–13,960], p = 0.002), while shorter delays (1–10 minutes) were significant predictors of errors at the ‘Incision’ event (OR = 12.7, 95% CI [2.8–57.0], p < 0.001). No significant predictors were identified for the ‘In-OR,’ ‘Closure,’ or ‘Out of OR’ events (*p* > 0.05).

The proportion of delays varied significantly between service lines, with the lowest percentage observed in Gastroenterology (37.21%) and the highest in Orthopedics (71.19%), followed closely by Neurosurgery (69.49%). Dental and Plastic Surgery also showed high delay rates at 58.62% and 45.76%, respectively. The average length of delays ranged from as low as 7.10 min in Otolaryngology and 8.24 min in Gastroenterology to 16.65 min in General Surgery, and 19.39 min in both Orthopedics and Multi-Panel (cases involving multiple service lines). Neurosurgery exhibited the longest average delay at 24.62 min (Table 2).

**Table 2:** Results by service Line. Documentation accuracy and delays in time-stamping by surgical service line. Metrics include the total number of events, the percentage of events correctly recorded, delays (frequency and average duration in minutes), and error rates

Metric/Service	DEN	OTO	GEN	GI	NEURO	OPH	ORTH	PLA	URO	M.*P*.
Events	29	356	193	129	59	119	59	59	102	112
Events correctly recorded	26	310	155	117	42	101	39	53	84	83
% Correct	89.66%	87.08%	80.31%	90.70%	71.19%	84.87%	66.10%	89.83%	82.35%	74.11%
Delays	17	182	108	48	41	54	42	27	49	62
% with delay	58.62%	51.12%	55.96%	37.21%	69.49%	45.38%	71.19%	45.76%	48.04%	55.36%
Average Length of Delay in Minutes (Standard Deviation)	11.72 (3.52)	7.10 (7.30)	16.65 (13.07)	8.24 (3.25)	24.62 (17.05)	11.90 (9.57)	19.39 (16.06)	10.20 (7.27)	10.80 (10.59)	19.39 (15.40)
Error Rate	10.34%	12.92%	19.69%	9.30%	28.81%	15.13%	33.90%	10.17%	17.65%	25.89%

There was a clear relationship between procedure complexity and error rates. While Gastroenterology had the lowest error rate (9.30%), Orthopedics again had the highest (33.90%), with Neurosurgery (28.81%) and Multi-Panel cases (25.89%) also exhibiting elevated error rates. These results underscore that as procedure complexity increases—whether due to equipment requirements, larger teams, or intricate workflows—the error rate rises.

## Discussion

This study demonstrates a strong and clinically meaningful relationship between documentation delays and EHR timestamp error rates in the operating room. These findings reinforce the broader human factors literature indicating that the accuracy of retrospective documentation deteriorates as cognitive load increases and memory becomes less reliable, and also align with and extend prior work in anesthesiology, where high documentation error rates have also been attributed to cognitive load, multitasking, and medico-legal motives that shape documentation priorities [14, 15]. Previous anesthesiology studies also report an association between delay and error consistent with our findings [7, 16]. These parallels suggest that the limitations of human-generated timestamps are not isolated to surgical nursing workflows but may represent a broader, cross-disciplinary constraint in the perioperative environment.

The volume of charting tasks required for each surgery is substantial. Our ORs perform over 10,000 cases annually resulting in approximately 2.53 million nurse charting tasks per year. This workload, combined with the complex environment of the OR, places additional stress on staff likely increasing error rates. Notably, our data suggest that although reducing the number of charting tasks may help unburden staff, it is unlikely to substantially reduce overall error rates if delays in documentation persist.

The consequences of timestamp inaccuracy extend beyond simple chronological misalignment. While major financial failures attributable solely to OR timestamping errors have not been documented, the cumulative effect of inaccurate data can meaningfully impact quality assurance, retrospective analyses, and root cause investigations. High‑quality organizations employ audit mechanisms to mitigate such risks, yet these processes require additional labor and infrastructure and cannot fully compensate for source data inaccuracies. Moreover, errors in EHR timestamping may limit the identification of causal and contributing factors during root cause analysis, thereby hindering the development of targeted mitigation strategies to reduce risk and prevent harm to patients.

Looking forward, demographics shifts and workforce shortages will place additional strain on manual documentation processes. With the Baby Boomer generation expected to be fully retired by 2031, healthcare utilization is projected to increase, including rising surgical volumes [17, 18]. At the same time, the healthcare is facing the mass retirement of the current nursing workforce, exacerbating the shortage of skilled healthcare professionals [19–21], which underscores the urgency of adopting technologies capable of reducing reliance on human-entered timestamps.

Several maturing technologies offer promising paths. Real-time locating systems (RTLS) using infrared, RFID, or hybrid sensors can automatically detect patient entry, exit, and bed movement. Integration of device-generated events such as ventilator on/off timestamps, anesthesia machine states, medication documentation and instrument tray RFID scans could further eliminate manual steps [22]. Computer vision systems may eventually identify key procedural milestones such as incision and closure with high temporal precision. Finally, AI-based documentation assistants, as recently demonstrated in anesthesiology, show early promise in capturing events automatically through multimodal signal fusion [11].

### Limitations

This study has several limitations. First, it was conducted at a single institution, which may limit generalizability of the findings to other hospitals or surgical settings with different workflows, staffing models, or documentation systems. Second, while we identified associations between environmental complexity, documentation delays, and error rates, these results are observational and cannot establish causality. The presence of additional staff, larger operating rooms, or multiple service lines may coincide with other unmeasured factors such as procedural difficulty, fatigue, or case urgency, which could also influence error rates. Finally, cognitive load was inferred from environmental factors and charting delays rather than being measured directly. Although previous work [5] has shown that complex environments increase the likelihood of communication failures and errors, our study did not assess workload or memory strain at the individual level.

## Conclusion

Documentation errors in the EHR during surgery exceed those for similar tasks in high-end manufacturing indicating that the OR is a high-risk environment for errors. New strategies and technologies to limit errors in human data entry into the EHR will be needed if this problem is to be corrected. Future research should focus on validating these technologies in live surgical environments, assessing their interoperability with EHR systems, and determining which events can be reliably automated without human confirmation. Finally, transitioning from human-based to technology-assisted informatics may represent the most effective strategy for improving timestamp reliability, reducing cognitive burden on clinical staff, and enabling high-fidelity perioperative analytics.

## Supplementary Information

Below is the link to the electronic supplementary material.

Supplementary Material 1

## Data Availability

Data from this study we be made available upon reasonable request.
