# Correction: The Limits of Humans in Data Gathering: Documentation Error Rates in the Electronic Health Record in the Operating Room

**DOI:** 10.1007/s10916-026-02396-z

**Published:** 2026-04-27

**Authors:** Andrew R. Bradley, Abner Barbosa, Logan Younk, Naila Rocha, Peter F. Nichol

**Affiliations:** 1 Department of Surgery, University of Wisconsin School of MedicineUniversity of Wisconsin School of Medicine, and Public Health, H4/746 CSC 600 Highland Ave, Madison, WI 53792 USA; 2 Department of Animal and Dairy Sciences, University of Wisconsin- Madison, Madison, WI USA

**Correction: Journal of Medical Systems (2026) 50:17**

10.1007/s10916-026-02346-9

The originally published version of this article contained mistakes and the authors would like to correct it.

A typo was found in Figure 1 where "Length" was mistakenly spelled as "Lenght".

The original article has been corrected.

