# Supplementary material for: The Limits of Humans in Data Gathering: Documentation Error Rates in the Electronic Health Record in the Operating Room
Source: J Med Syst. 2026 Feb 9;50(1):17. doi: 10.1007/s10916-026-02346-9 (PMC12883500; doi:10.1007/s10916-026-02346-9)
Supplement: Supplementary file 1 — Supplementary Material 1 [file 10916_2026_2346_MOESM1_ESM.docx]

September 12, 2025

Re: Revisions and response to the reviewers from previously reviewed paper

Dear Dr. **Simpao,**

We thank the editor and reviewers for their careful reading of our manuscript and their constructive feedback. We have addressed each comment in detail below, indicating how we revised the manuscript accordingly. Additionally, a Limitations section has been added at the end of the Conclusion. This section discusses the single institution setting, observational design, potential unmeasured confounders, the indirect measurement of cognitive load and limited scope of documentation errors examined. This addition addresses broader concerns about interpretation and generalizability.

**1.** **Please justify why no nurses are included in the study nor the interpretation of the results.**
*Our nurses do not have research project time due to high case volume, staff turnover and the constrained labor market. We acknowledge the concern regarding the absence of nurse authorship or interpretive input. In this study, nurses participated only as the subjects of observation and were intentionally not involved in the interpretation of results to avoid introducing bias. The interpretation was instead carried out by the study designers and the statistical team, who analyzed the observed documentation patterns using pre-defined metrics. In the future, as we did in our previous studies, we will invite them to participate.*

**2.** **You alternate between “time stamping,” “time-stamping,” and “charting events.” Consider standardizing terminology throughout.**
*All terminology has been standardized throughout the manuscript. We now consistently use “time-stamping” as the preferred term, except where specific variation was necessary to preserve accuracy in referencing literature.*

**3.** **The discussion sometimes blurs whether time stamping is intrinsically more complex than assumed or whether environmental complexity makes it so. You could make this dichotomy more explicit and indicate which you believe is the stronger driver.**
*We thank the reviewer for pointing out this important conceptual distinction. We have revised the Discussion to explicitly address both interpretations. Specifically, we note that time-stamping, although categorized as a “simple routine” task in human factors models, may function as a more complex task in practice due to the layered and dynamic environment of the operating room. Our findings suggest that environmental complexity is the stronger driver of error rates, although we cannot fully exclude the possibility that EHR time-stamping itself is more complex than assumed. This distinction has been clarified in the revised text.*

**4.** **Some p-values are presented without confidence intervals or effect sizes. Including these where relevant (especially in regression results) would make the findings more interpretable. Please include them.**
*We have carefully reviewed the statistical reporting. Confidence intervals and effect sizes have been added where relevant. Specifically:*

*Regression models: We now report beta estimates, R², and 95% confidence intervals.*

*Logistic regression: Odds ratios and 95% confidence intervals were added.*

*Chi-square tests: We clarified that these analyses do not yield confidence intervals but have explicitly labeled them as chi-square results.*

**5.** **You missed a reference and should remove the stray “(REF)” in the significance section.**
*We thank the reviewer for catching this oversight. We have replaced the placeholder with appropriate references.*

**6.** **Please define “M.P.” (multi-panel) when first used in Results, not just in Methods.**
*This has been corrected. We now define M.P. as multi-panel cases at the first mention in the Results section.*

**7.** **One can reasonable ask should be: One can reasonably ask.**
*The error has been corrected.*

**8. Ethics statement.**

*This has been added in the Declarations Section at the end of the manuscript before the references*

Warmly and respectfully,

Peter Nichol MD/PhD

Associate Professor of Surgery

University of Wisconsin School of Medicine and Public Health

H4/746 CSC

600 Highland Ave.

Madison, WI 53792
